# Crystal structure and Hirshfeld surface analysis and of 2-ammoniumylmeth­yl-1*H*-benzimidazol-3-ium chloride monohydrate

**DOI:** 10.1107/S205698901801335X

**Published:** 2018-09-28

**Authors:** Pinar Sen, Sevgi Kansiz, Necmi Dege, S. Zeki Yildiz, Galyna G. Tsapyuk

**Affiliations:** a Uskudar University, Faculty of Engineering and Natural Sciences, Department of Forensic Science, 34662, Istanbul, Turkey; b Ondokuz Mayıs University, Faculty of Arts and Sciences, Department of Physics, 55139, Kurupelit, Samsun, Turkey; c Sakarya University, Faculty of Arts and Sciences, Department of Chemistry, 54187 Sakarya, Turkey; d Department of General Chemistry, O. O. Bohomolets National Medical University, Shevchenko Blvd. 13, 01601 Kiev, Ukraine

**Keywords:** crystal structure, imidazol, ethanaminium, ethanaminium chloride, Hirshfeld surface

## Abstract

The asymmetric unit of the title compound, C_8_H_11_N_3_^2+^·2Cl^−^·H_2_O, contains three organic cations, six chloride anions and three water mol­ecules of crystallization, which are connected by extensive hydrogen-bonding inter­actions into a three-dimensional supra­molecular architecture. Hirshfeld surface analysis and two-dimensional fingerprint plots indicate that the most important contributions to the crystal packing are from H⋯H (37.4%), Cl⋯H/H⋯Cl (35.5%), C⋯H/H⋯C (9.5%) and C⋯C (6.9%) inter­actions.

## Abstract 2

In the crystal, the crystal packing is ordered *via* synergetic contributions from N—H⋯Cl, O—H⋯Cl and N—H⋯O hydrogen bonds, which together assemble the cations and anions into a three-dimensional framework.

## Chemical context   

Heterocyclic compounds containing nitro­gen such as benzimidazoles and their derivatives have attracted attention because of their medicinal applications as anti­ulcer, anti­cancer, anti­fungal, anti­mycobacterial and anti-inflammatory agents (El-masry *et al.*, 2000 ▸). Besides being important pharma­cophores, in particular amine-substituted benzimidazoles are good inter­mediates for the synthesis of different organic compounds (Maurya *et al.*, 2007 ▸). General methods for the preparation of benzimidazoles involve the reaction of *o*-phenyl­enedi­amine and carb­oxy­lic acid or its derivatives under harsh dehydrating conditions or with aldehydes followed by oxidation (Peng *et al.*, 2014 ▸).
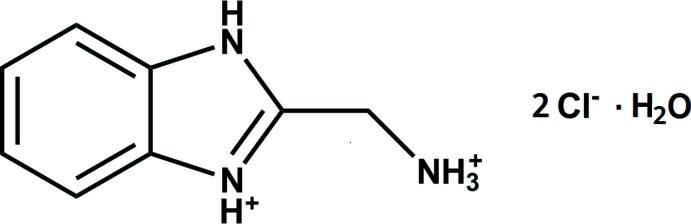


We report herein the compound 2-amino­methyl­benzimidazole di­hydro­chloride (ambmz·2HCl) prepared as described previously (Wu *et al.*, 2008 ▸)

## Structural commentary   

**Figure 1:**
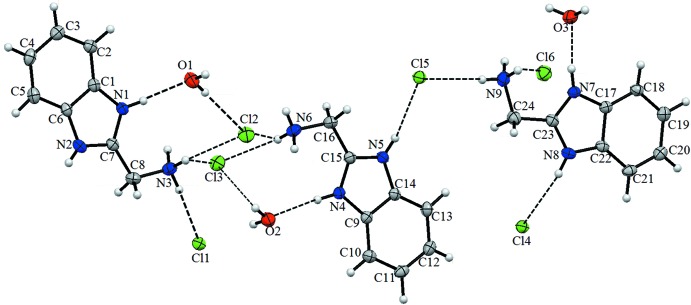
The mol­ecular structure of the title compound, showing the atom labelling. Displacement ellipsoids are drawn at the 20% probability level.

The asymmetric unit of the title compound contains three organic cations, six chloride anions and three water mol­ecules of crystallization, which are connected by O—H⋯Cl, N—H⋯O and N—H⋯Cl hydrogen bonds (Fig. 1 ▸). The r.m.s. deviations of the benzimidazolium ring systems are 0.0085 Å for N1/N2/C1–C7, 0.0076 Å for N4/N5/C9–C15, 0.0063 Å for N7/N8/C17–C23 with maximum deviations from planarity of 0.0169 (13) Å for atom C7, 0.0149 (13) Å for atom C15 and 0.0132 (13) Å for atom C23, respectively. The observed bond lengths are in good agreement with previously reported values (Cui, 2011 ▸).

## Supra­molecular features   

**Figure 2:**
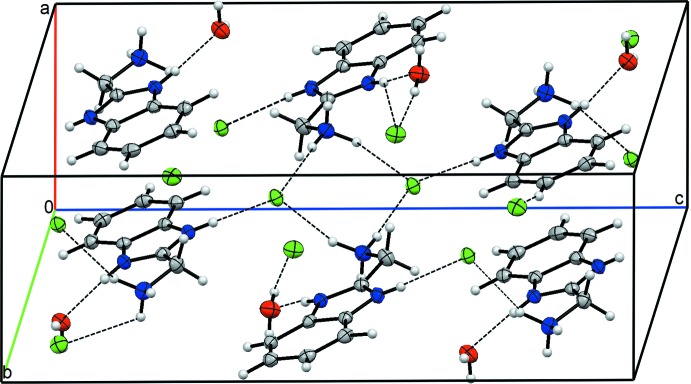
The view of the crystal packing of the title compound. Dashed lines denote the hydrogen bonds.

The crystal packing of the title compound features extensive hydrogen bonding (Table 1 ▸ and Fig. and 2) involving all three O atoms and all nine N atoms. N5—H5*A*⋯Cl5, N8—H8⋯Cl4, N2—H2⋯Cl1^ii^, N9—H9*C*⋯Cl5^vi^ and N6—H6*B*⋯Cl1^v^ hydrogen bonds link the ions into chains along the *c*-axis direction. These chains are linked by O–H⋯Cl and N—H⋯O hydrogen bonds, generating a three-dimensional network (Fig. 2 ▸).

## Hirshfeld surface analysis   

**Figure 3:**
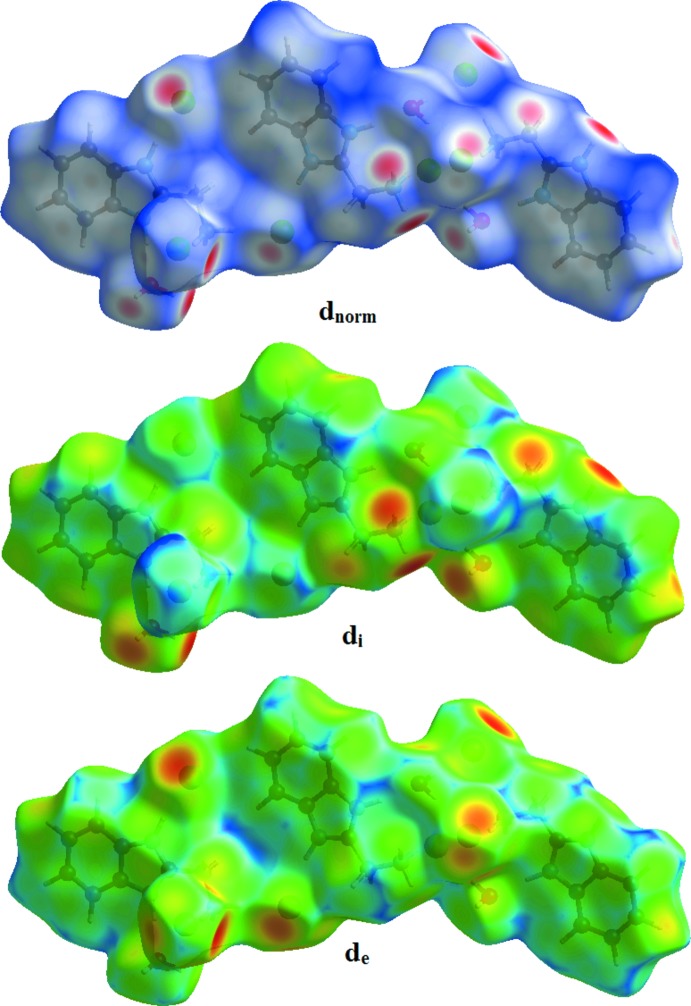
The Hirshfeld surface of the title compound mapped over *d*_norm_, *d*_i_ and *d*_e_.

**Figure 4:**
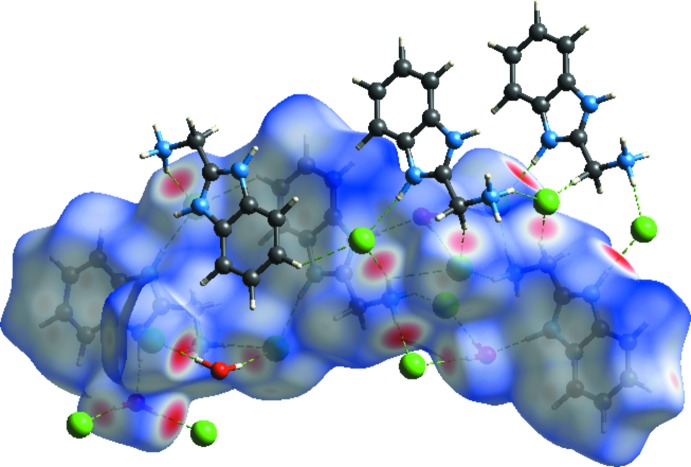
Hirshfeld surface mapped over *d*_norm_ to visualize the inter­molecular inter­actions of the title compound.

The Hirshfeld surface analysis was performed using *Crystal Explorer* (Turner *et al.*, 2017 ▸). The Hirshfeld surfaces, illus­trated in Fig. 3 ▸, and their associated two-dimensional fingerprint plots were used to qu­antify the various inter­molecular inter­actions in the synthesized complex. Red spots on the Hirshfeld surfaces indicate the inter­molecular contacts involved in strong hydrogen bonds and inter­atomic contacts (Gümüş *et al.*, 2018 ▸; Kansız *et al.*, 2018 ▸; Kansız & Dege, 2018 ▸). The red spots in Fig. 4 ▸ correspond to the H⋯Cl contacts resulting from the N—H⋯Cl and O—H⋯Cl hydrogen bonds. The Hirshfeld surfaces were mapped using a standard (high) surface resolution with the three-dimensional *d_norm_* surfaces mapped over a fixed colour scale of −0.518 (red) to 1.174 (blue) a.u..

**Figure 5:**
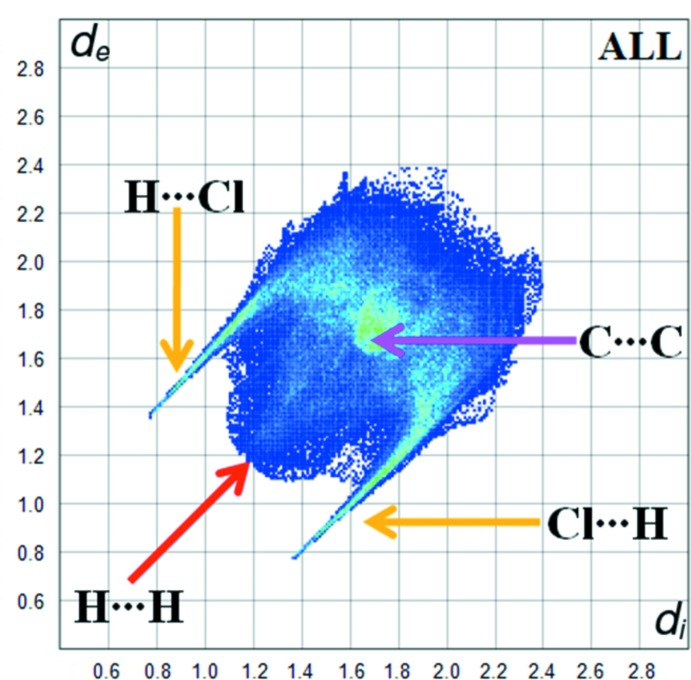
Fingerprint plot of all the contacts.

**Figure 6:**
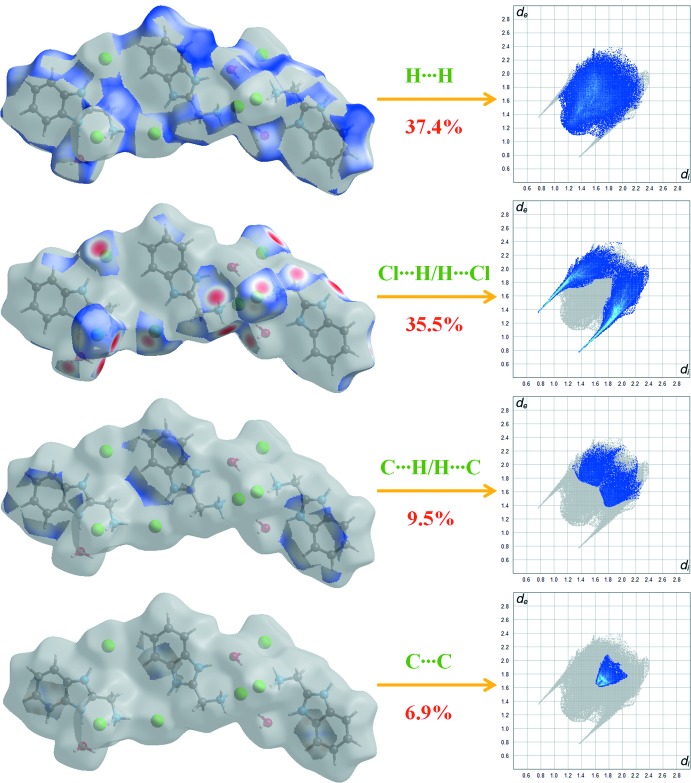
Two-dimensional fingerprint plots with a *d*_norm_ view of the H⋯H (37.4%), Cl⋯H/H⋯Cl (35.5%), C⋯H/H⋯C (9.5%) and C⋯C (6.9%) contacts in the title compound.

Fig. 5 ▸ shows the two-dimensional fingerprint plot of all the contacts contributing to the Hirshfeld surface represented in normal mode. Fig. 6 ▸ shows the two-dimensional fingerprint plots of the (*d*_i_, *d*_e_) points associated with various atoms. H⋯H contacts contribute 37.4% to the Hirshfeld surface. The graph for Cl⋯H/H⋯Cl shows the contacts between the chlorine atoms inside the Hirshfeld surface and the hydrogen atoms outside the surface and *vice versa*, and has two symmetrical wings on the left and right sides (35.5%). Further, there are C⋯H/H⋯C (9.5%), C⋯C (6.9%), O⋯H/H⋯O (4.1%) and N⋯H/H⋯N (3.4%) contacts.

## Synthesis and crystallization   

**Figure 7:**
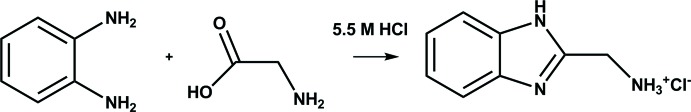
The synthesis of the title compound.

*o*-Phenyl­enedi­amine (10.8 g, 99.87 mmol) and glycine (10.00 g, 133.2 mmol) were dissolved in 5.5 *M* HCl (150 mL) . The reaction mixture was purged by argon at room temperature and heated up to reflux temperature for 12 h. The reaction was monitored by TLC. After completion of the reaction, the mixture was concentrated to 50 mL and kept at 269 K for 2 d. The crystals were filtered off and washed twice with acetone and dried to give the desired product (Fig. 7 ▸).

## Refinement   

Crystal data, data collection and structure refinement details are summarized in Table 2 ▸. C-bound H atoms were positioned geometrically with C—H distances of 0.93–0.97 Å. and refined as riding, with *U*_iso_(H) = 1.2*U*_eq_(C). N-bound H atoms were located in difference-Fourier maps and refined isotropically. The coordinates of the water H atoms were determined from a difference-Fourier map and refined isotropically subject to a restraint of O—H = 0.82 (4) Å.

## Supplementary Material

Crystal structure: contains datablock(s) I. DOI: 10.1107/S205698901801335X/xu5941sup1.cif

Structure factors: contains datablock(s) I. DOI: 10.1107/S205698901801335X/xu5941Isup2.hkl

Supporting information file. DOI: 10.1107/S205698901801335X/xu5941Isup3.cml

CCDC reference: 1868580

Additional supporting information:  crystallographic information; 3D view; checkCIF report

## Figures and Tables

**Table 1:** Hydrogen-bond geometry (Å, °)

*D*—H⋯*A*	*D*—H	H⋯*A*	*D*⋯*A*	*D*—H⋯*A*
O1—H1*A*⋯Cl4^i^	0.82 (2)	2.63 (2)	3.4431 (15)	168 (3)
O1—H2*B*⋯Cl2	0.81 (2)	2.36 (2)	3.1598 (18)	169 (3)
O2—H2*C*⋯Cl3	0.82 (2)	2.33 (2)	3.1244 (17)	165 (2)
O2—H2*D*⋯Cl1	0.81 (1)	2.64 (1)	3.4492 (15)	171 (3)
O3—H3*D*⋯Cl5^ii^	0.80 (2)	2.69 (2)	3.4586 (15)	164 (3)
O3—H3*E*⋯Cl6^ii^	0.82 (2)	2.32 (2)	3.1364 (18)	173 (3)
N1—H1⋯O1	0.83 (2)	1.92 (2)	2.746 (2)	174 (2)
N2—H2⋯Cl1^iii^	0.71 (2)	2.44 (2)	3.1519 (17)	175 (2)
N3—H3⋯Cl3	0.88 (3)	2.30 (3)	3.105 (2)	153 (2)
N3—H3*A*⋯Cl1	0.86 (4)	2.26 (3)	3.119 (2)	173 (3)
N3—H3*B*⋯Cl4^iv^	0.99 (3)	2.33 (2)	3.267 (2)	156.8 (19)
N4—H4*A*⋯O2	0.85 (2)	1.91 (2)	2.754 (2)	171 (2)
N5—H5*A*⋯Cl5	0.83 (2)	2.31 (2)	3.1205 (17)	165 (2)
N6—H6*A*⋯Cl2	0.87 (3)	2.33 (3)	3.107 (2)	150 (2)
N6—H6*B*⋯Cl1^v^	0.98 (3)	2.36 (2)	3.287 (2)	158.2 (18)
N6—H6*C*⋯Cl4^i^	0.79 (3)	2.35 (4)	3.1347 (19)	176 (3)
N7—H7⋯O3	0.85 (3)	1.89 (3)	2.742 (2)	175 (3)
N8—H8⋯Cl4	0.81 (2)	2.31 (2)	3.1049 (17)	172 (2)
N9—H9*A*⋯Cl6	0.83 (3)	2.35 (3)	3.100 (2)	151 (2)
N9—H9*B*⋯Cl5	0.80 (3)	2.32 (2)	3.120 (2)	176 (2)
N9—H9*C*⋯Cl5^vi^	0.99 (3)	2.34 (3)	3.270 (2)	157 (2)
C4—H4⋯Cl1^i^	0.92 (2)	2.81 (2)	3.535 (2)	136.7 (16)
C8—H8*B*⋯Cl3^vii^	0.93 (3)	2.65 (3)	3.544 (2)	162 (2)

Symmetry codes: (i) 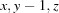; (ii) 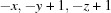; (iii) 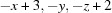; (iv) 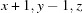; (v) 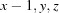; (vi) 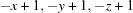; (vii) 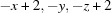.

**Table 2:** Experimental details

Crystal data	
Chemical formula	C_8_H_11_N_3_^+^·2Cl^−^·H_2_O·
*M*_r_	238.11
Crystal system, space group	Triclinic, *P* 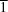
Temperature (K)	296
*a*, *b*, *c* (Å)	6.9340 (4), 12.1198 (7), 19.2128 (11)
α, β, γ (°)	99.859 (5), 90.647 (5), 90.247 (5)
*V* (Å^3^)	1590.64 (16)
*Z*	6
Radiation type	Mo *K*α
μ (mm^−1^)	0.58
Crystal size (mm)	0.57 × 0.50 × 0.46

Data collection	
Diffractometer	Stoe IPDS 2
Absorption correction	Integration (*X-RED32*; Stoe & Cie, 2002 ▸)
*T*_min_, *T*_max_	0.788, 0.828
No. of measured, independent and observed [*I* > 2σ(*I*)] reflections	16045, 6254, 5000
*R*_int_	0.064
(sin θ/λ)_max_ (Å^−1^)	0.617

Refinement	
*R*[*F* ^2^ > 2σ(*F* ^2^)], *wR*(*F* ^2^), *S*	0.035, 0.092, 0.96
No. of reflections	6254
No. of parameters	536
No. of restraints	9
H-atom treatment	All H-atom parameters refined
Δρ_max_, Δρ_min_ (e Å^−3^)	0.34, −0.28

Computer programs: *X-AREA* and *X-RED32* (Stoe & Cie, 2002 ▸), *SHELXL2017/1* (Sheldrick, 2015 ▸), *ORTEP-3 for Windows* and *WinGX* (Farrugia, 2012 ▸) and *PLATON* (Spek, 2009 ▸).

## supplementary crystallographic information

### Crystal data

**Table:**

C_8_H_11_N_3_^+^·2Cl^−^·H_2_O·	*Z* = 6
*M**_r_* = 238.11	*F*(000) = 744
Triclinic, *P*1	*D*_x_ = 1.491 Mg m^−^^3^
*a* = 6.9340 (4) Å	Mo *K*α radiation, λ = 0.71073 Å
*b* = 12.1198 (7) Å	Cell parameters from 18110 reflections
*c* = 19.2128 (11) Å	θ = 1.7–27.4°
α = 99.859 (5)°	µ = 0.58 mm^−^^1^
β = 90.647 (5)°	*T* = 296 K
γ = 90.247 (5)°	Prism, brown
*V* = 1590.64 (16) Å^3^	0.57 × 0.50 × 0.46 mm

### Data collection

**Table:**

Stoe IPDS 2 diffractometer	6254 independent reflections
Radiation source: sealed X-ray tube, 12 x 0.4 mm long-fine focus	5000 reflections with *I* > 2σ(*I*)
Detector resolution: 6.67 pixels mm^-1^	*R*_int_ = 0.064
rotation method scans	θ_max_ = 26.0°, θ_min_ = 1.7°
Absorption correction: integration (X-RED32; Stoe & Cie, 2002)	*h* = −8→8
*T*_min_ = 0.788, *T*_max_ = 0.828	*k* = −14→14
16045 measured reflections	*l* = −23→23

### Refinement

**Table:**

Refinement on *F*^2^	Hydrogen site location: difference Fourier map
Least-squares matrix: full	All H-atom parameters refined
*R*[*F*^2^ > 2σ(*F*^2^)] = 0.035	*w* = 1/[σ^2^(*F*_o_^2^) + (0.056*P*)^2^] where *P* = (*F*_o_^2^ + 2*F*_c_^2^)/3
*wR*(*F*^2^) = 0.092	(Δ/σ)_max_ = 0.001
*S* = 0.96	Δρ_max_ = 0.34 e Å^−^^3^
6254 reflections	Δρ_min_ = −0.28 e Å^−^^3^
536 parameters	Extinction correction: SHELXL-2017/1 (Sheldrick 2015), Fc^*^=kFc[1+0.001xFc^2^λ^3^/sin(2θ)]^-1/4^
9 restraints	Extinction coefficient: 0.0327 (17)

### Special details

**Table:**

Geometry. All esds (except the esd in the dihedral angle between two l.s. planes) are estimated using the full covariance matrix. The cell esds are taken into account individually in the estimation of esds in distances, angles and torsion angles; correlations between esds in cell parameters are only used when they are defined by crystal symmetry. An approximate (isotropic) treatment of cell esds is used for estimating esds involving l.s. planes.

### Fractional atomic coordinates and isotropic or equivalent isotropic displacement parameters (Å^2^)

**Table:**

	*x*	*y*	*z*	*U*_iso_*/*U*_eq_	
Cl1	1.44783 (7)	0.25544 (4)	0.93231 (2)	0.04012 (12)	
Cl4	0.55220 (7)	0.93572 (4)	0.73215 (2)	0.03912 (12)	
Cl5	0.44034 (7)	0.39635 (4)	0.59956 (2)	0.04089 (13)	
Cl3	0.85067 (7)	0.04098 (4)	0.91238 (3)	0.04840 (14)	
Cl6	−0.13546 (8)	0.60301 (5)	0.58937 (3)	0.05432 (15)	
Cl2	1.13465 (8)	0.15568 (5)	0.74387 (3)	0.05337 (15)	
O2	0.9557 (2)	0.29533 (13)	0.93050 (9)	0.0492 (3)	
O1	1.0439 (2)	−0.10288 (14)	0.73035 (9)	0.0517 (4)	
N4	0.7622 (2)	0.41261 (12)	0.84094 (8)	0.0332 (3)	
O3	0.0525 (2)	0.64579 (14)	0.39493 (9)	0.0553 (4)	
N7	0.2335 (2)	0.81071 (13)	0.48888 (8)	0.0333 (3)	
N5	0.6592 (2)	0.46067 (13)	0.74372 (9)	0.0347 (3)	
N1	1.2273 (2)	−0.21584 (13)	0.82407 (8)	0.0334 (3)	
N2	1.3362 (2)	−0.25722 (14)	0.92226 (9)	0.0376 (4)	
N8	0.3400 (2)	0.90994 (13)	0.58704 (9)	0.0358 (3)	
N6	0.7067 (3)	0.17781 (15)	0.79023 (10)	0.0427 (4)	
N9	0.2868 (3)	0.60170 (15)	0.53791 (10)	0.0447 (4)	
N3	1.2803 (3)	0.02017 (15)	0.86850 (10)	0.0421 (4)	
C14	0.7037 (2)	0.55975 (14)	0.78881 (9)	0.0314 (4)	
C22	0.2942 (2)	0.98527 (15)	0.54300 (9)	0.0323 (4)	
C9	0.7708 (2)	0.52904 (14)	0.85148 (9)	0.0315 (4)	
C15	0.6935 (2)	0.37525 (15)	0.77671 (9)	0.0335 (4)	
C1	1.2214 (2)	−0.33227 (14)	0.81587 (9)	0.0324 (4)	
C17	0.2255 (2)	0.92225 (14)	0.48021 (9)	0.0319 (4)	
C10	0.8268 (3)	0.60738 (16)	0.90934 (10)	0.0369 (4)	
C18	0.1652 (2)	0.97150 (16)	0.42385 (10)	0.0355 (4)	
C7	1.2981 (2)	−0.17423 (15)	0.88773 (9)	0.0339 (4)	
C2	1.1642 (3)	−0.41361 (16)	0.75996 (10)	0.0377 (4)	
C6	1.2930 (2)	−0.35839 (15)	0.87927 (10)	0.0349 (4)	
C13	0.6926 (3)	0.67112 (16)	0.78093 (11)	0.0385 (4)	
C23	0.3047 (2)	0.80652 (15)	0.55283 (9)	0.0342 (4)	
C20	0.2469 (3)	1.15029 (16)	0.49646 (11)	0.0392 (4)	
C21	0.3055 (3)	1.10154 (16)	0.55279 (11)	0.0382 (4)	
C12	0.7490 (3)	0.74911 (16)	0.83844 (11)	0.0394 (4)	
C5	1.3092 (3)	−0.46915 (17)	0.88878 (11)	0.0421 (4)	
C19	0.1785 (3)	1.08684 (16)	0.43313 (10)	0.0389 (4)	
C4	1.2532 (3)	−0.54982 (17)	0.83351 (12)	0.0443 (5)	
C11	0.8145 (3)	0.71817 (16)	0.90146 (11)	0.0401 (4)	
C8	1.3424 (3)	−0.05475 (17)	0.91637 (11)	0.0414 (4)	
C16	0.6469 (3)	0.25681 (17)	0.74466 (11)	0.0428 (4)	
C3	1.1819 (3)	−0.52313 (17)	0.77020 (12)	0.0423 (4)	
C24	0.3525 (3)	0.70519 (18)	0.58323 (12)	0.0458 (5)	
H2	1.384 (3)	−0.2526 (17)	0.9558 (11)	0.028 (5)*	
H18	0.111 (3)	0.9325 (18)	0.3840 (12)	0.044 (6)*	
H10	0.873 (3)	0.5879 (16)	0.9522 (11)	0.033 (5)*	
H2A	1.108 (3)	−0.3947 (17)	0.7161 (11)	0.039 (5)*	
H11	0.854 (3)	0.7742 (19)	0.9433 (11)	0.046 (6)*	
H19	0.136 (3)	1.1218 (17)	0.3927 (11)	0.039 (5)*	
H20	0.255 (3)	1.223 (2)	0.5012 (12)	0.048 (6)*	
H13	0.649 (3)	0.6891 (17)	0.7361 (11)	0.039 (5)*	
H12	0.740 (3)	0.816 (2)	0.8342 (12)	0.050 (6)*	
H21	0.350 (3)	1.1439 (19)	0.5987 (12)	0.047 (6)*	
H5	1.355 (3)	−0.4842 (19)	0.9333 (12)	0.050 (6)*	
H4	1.264 (3)	−0.6238 (19)	0.8385 (11)	0.040 (5)*	
H5A	0.608 (3)	0.4556 (18)	0.7041 (12)	0.041 (6)*	
H1	1.179 (3)	−0.179 (2)	0.7956 (13)	0.055 (7)*	
H4A	0.811 (3)	0.372 (2)	0.8683 (12)	0.052 (6)*	
H8	0.393 (3)	0.9236 (17)	0.6251 (12)	0.036 (5)*	
H24A	0.291 (3)	0.710 (2)	0.6289 (13)	0.058 (7)*	
H24B	0.491 (5)	0.697 (3)	0.5884 (17)	0.101 (10)*	
H7	0.183 (4)	0.757 (2)	0.4601 (14)	0.063 (7)*	
H9A	0.167 (4)	0.600 (2)	0.5354 (13)	0.058 (7)*	
H16A	0.518 (4)	0.253 (2)	0.7334 (15)	0.076 (8)*	
H9B	0.325 (4)	0.551 (2)	0.5559 (13)	0.055 (7)*	
H9C	0.336 (4)	0.593 (2)	0.4891 (16)	0.080 (9)*	
H16B	0.709 (3)	0.2385 (19)	0.7019 (13)	0.052 (6)*	
H3C	1.143 (3)	−0.581 (2)	0.7340 (12)	0.050 (6)*	
H8A	1.472 (4)	−0.045 (2)	0.9269 (13)	0.061 (7)*	
H8B	1.282 (4)	−0.035 (2)	0.9596 (14)	0.066 (7)*	
H6A	0.831 (4)	0.176 (2)	0.7947 (13)	0.061 (8)*	
H6B	0.660 (3)	0.194 (2)	0.8388 (14)	0.059 (7)*	
H6C	0.667 (4)	0.118 (3)	0.7738 (15)	0.065 (8)*	
H2C	0.920 (3)	0.2322 (16)	0.9337 (14)	0.063 (8)*	
H2D	1.072 (2)	0.289 (2)	0.9267 (17)	0.098 (12)*	
H3A	1.316 (4)	0.087 (3)	0.8871 (16)	0.086 (10)*	
H3B	1.330 (3)	0.000 (2)	0.8197 (14)	0.060 (7)*	
H3	1.154 (4)	0.022 (2)	0.8652 (12)	0.056 (7)*	
H3D	−0.062 (3)	0.649 (3)	0.3941 (19)	0.106 (12)*	
H3E	0.082 (4)	0.5804 (16)	0.3962 (15)	0.071 (9)*	
H1A	0.926 (3)	−0.102 (3)	0.7339 (18)	0.100 (12)*	
H2B	1.076 (4)	−0.0391 (16)	0.7286 (15)	0.069 (9)*	

### Atomic displacement parameters (Å^2^)

**Table:**

	*U*^11^	*U*^22^	*U*^33^	*U*^12^	*U*^13^	*U*^23^
Cl1	0.0439 (2)	0.0399 (2)	0.0360 (2)	0.00037 (18)	−0.00373 (18)	0.00530 (18)
Cl4	0.0430 (2)	0.0388 (2)	0.0348 (2)	−0.00156 (18)	−0.00346 (18)	0.00436 (18)
Cl5	0.0447 (3)	0.0395 (2)	0.0378 (2)	−0.00330 (18)	−0.00542 (18)	0.00547 (18)
Cl3	0.0486 (3)	0.0458 (3)	0.0518 (3)	−0.0026 (2)	0.0038 (2)	0.0112 (2)
Cl6	0.0503 (3)	0.0508 (3)	0.0615 (3)	0.0005 (2)	−0.0013 (2)	0.0085 (2)
Cl2	0.0491 (3)	0.0519 (3)	0.0600 (3)	−0.0049 (2)	−0.0007 (2)	0.0126 (2)
O2	0.0490 (9)	0.0458 (9)	0.0538 (9)	−0.0016 (7)	−0.0076 (7)	0.0121 (7)
O1	0.0475 (9)	0.0546 (9)	0.0566 (9)	−0.0045 (7)	−0.0098 (7)	0.0209 (8)
N4	0.0346 (7)	0.0320 (7)	0.0337 (8)	0.0003 (6)	−0.0023 (6)	0.0071 (6)
O3	0.0496 (9)	0.0456 (9)	0.0652 (10)	0.0011 (7)	−0.0110 (8)	−0.0056 (7)
N7	0.0323 (7)	0.0331 (8)	0.0331 (8)	−0.0025 (6)	−0.0008 (6)	0.0017 (6)
N5	0.0336 (8)	0.0387 (8)	0.0314 (8)	0.0013 (6)	−0.0032 (6)	0.0047 (6)
N1	0.0317 (7)	0.0359 (8)	0.0341 (8)	−0.0008 (6)	−0.0022 (6)	0.0106 (6)
N2	0.0333 (8)	0.0494 (10)	0.0314 (9)	−0.0017 (7)	−0.0053 (7)	0.0107 (7)
N8	0.0342 (8)	0.0420 (9)	0.0298 (8)	−0.0059 (6)	−0.0038 (6)	0.0028 (6)
N6	0.0505 (11)	0.0310 (9)	0.0451 (10)	−0.0057 (8)	0.0012 (8)	0.0025 (7)
N9	0.0501 (11)	0.0375 (9)	0.0485 (11)	0.0051 (8)	0.0010 (8)	0.0128 (8)
N3	0.0469 (10)	0.0358 (9)	0.0426 (10)	−0.0042 (7)	0.0018 (8)	0.0034 (7)
C14	0.0264 (8)	0.0346 (9)	0.0331 (9)	0.0007 (6)	0.0018 (7)	0.0052 (7)
C22	0.0265 (8)	0.0378 (9)	0.0315 (9)	−0.0035 (7)	0.0011 (7)	0.0027 (7)
C9	0.0275 (8)	0.0312 (8)	0.0357 (9)	−0.0003 (6)	0.0023 (7)	0.0054 (7)
C15	0.0295 (8)	0.0347 (9)	0.0353 (9)	0.0001 (7)	0.0003 (7)	0.0029 (7)
C1	0.0266 (8)	0.0355 (9)	0.0361 (9)	−0.0010 (7)	0.0026 (7)	0.0093 (7)
C17	0.0274 (8)	0.0337 (9)	0.0334 (9)	−0.0023 (6)	0.0034 (7)	0.0019 (7)
C10	0.0349 (9)	0.0406 (10)	0.0343 (9)	−0.0015 (7)	0.0003 (7)	0.0042 (8)
C18	0.0306 (8)	0.0423 (10)	0.0320 (9)	−0.0003 (7)	0.0002 (7)	0.0016 (8)
C7	0.0287 (8)	0.0391 (9)	0.0343 (9)	−0.0018 (7)	0.0006 (7)	0.0073 (7)
C2	0.0319 (9)	0.0440 (10)	0.0371 (10)	−0.0025 (7)	0.0017 (7)	0.0066 (8)
C6	0.0269 (8)	0.0417 (10)	0.0378 (9)	0.0014 (7)	0.0008 (7)	0.0112 (8)
C13	0.0334 (9)	0.0399 (10)	0.0442 (11)	0.0032 (7)	0.0036 (8)	0.0130 (8)
C23	0.0291 (8)	0.0384 (9)	0.0353 (9)	−0.0026 (7)	0.0008 (7)	0.0066 (7)
C20	0.0344 (9)	0.0343 (10)	0.0485 (11)	−0.0028 (8)	0.0075 (8)	0.0051 (8)
C21	0.0318 (9)	0.0384 (10)	0.0408 (10)	−0.0061 (7)	0.0020 (8)	−0.0030 (8)
C12	0.0359 (9)	0.0307 (9)	0.0519 (12)	0.0024 (7)	0.0068 (8)	0.0076 (8)
C5	0.0339 (9)	0.0470 (11)	0.0498 (12)	0.0041 (8)	0.0033 (8)	0.0202 (9)
C19	0.0349 (9)	0.0419 (10)	0.0412 (10)	0.0012 (7)	0.0053 (8)	0.0100 (8)
C4	0.0354 (10)	0.0389 (10)	0.0608 (13)	0.0044 (8)	0.0106 (9)	0.0146 (10)
C11	0.0351 (9)	0.0367 (10)	0.0457 (11)	−0.0036 (7)	0.0058 (8)	−0.0013 (8)
C8	0.0423 (11)	0.0437 (11)	0.0362 (10)	−0.0059 (8)	−0.0033 (9)	0.0017 (8)
C16	0.0494 (12)	0.0369 (10)	0.0398 (11)	−0.0013 (8)	−0.0073 (9)	−0.0001 (8)
C3	0.0349 (9)	0.0401 (10)	0.0509 (12)	−0.0025 (8)	0.0069 (8)	0.0044 (9)
C24	0.0478 (11)	0.0460 (11)	0.0456 (12)	−0.0031 (9)	−0.0062 (9)	0.0141 (9)

### Geometric parameters (Å, º)

**Table:**

O2—H2C	0.817 (16)	C22—C17	1.391 (2)
O2—H2D	0.812 (17)	C22—C21	1.391 (3)
O1—H1A	0.819 (17)	C9—C10	1.383 (3)
O1—H2B	0.810 (16)	C15—C16	1.494 (3)
N1—C7	1.328 (2)	C1—C2	1.381 (3)
N1—C1	1.393 (2)	C1—C6	1.396 (2)
N1—H1	0.83 (2)	C17—C18	1.384 (2)
N2—C7	1.322 (2)	C10—C11	1.380 (3)
N2—C6	1.386 (3)	C10—H10	0.95 (2)
N2—H2	0.71 (2)	C18—C19	1.381 (3)
N3—C8	1.461 (3)	C18—H18	0.91 (2)
N3—H3A	0.87 (4)	C7—C8	1.488 (3)
N3—H3B	0.99 (3)	C2—C3	1.381 (3)
N3—H3	0.88 (3)	C2—H2A	0.99 (2)
N4—C15	1.322 (2)	C6—C5	1.390 (3)
N4—C9	1.392 (2)	C13—C12	1.377 (3)
N4—H4A	0.85 (2)	C13—H13	0.97 (2)
N5—C15	1.324 (2)	C23—C24	1.484 (3)
N5—C14	1.386 (2)	C20—C21	1.377 (3)
N5—H5A	0.83 (2)	C20—C19	1.400 (3)
N6—C16	1.462 (3)	C20—H20	0.87 (2)
N6—H6A	0.86 (3)	C21—H21	0.99 (2)
N6—H6B	0.98 (3)	C12—C11	1.399 (3)
N6—H6C	0.79 (3)	C12—H12	0.83 (2)
O3—H3D	0.796 (17)	C5—C4	1.366 (3)
O3—H3E	0.822 (16)	C5—H5	0.96 (2)
N7—C23	1.328 (2)	C19—H19	0.99 (2)
N7—C17	1.391 (2)	C4—C3	1.397 (3)
N7—H7	0.85 (3)	C4—H4	0.92 (2)
N8—C23	1.332 (2)	C11—H11	0.99 (2)
N8—C22	1.383 (2)	C8—H8A	0.92 (3)
N8—H8	0.80 (2)	C8—H8B	0.93 (3)
N9—C24	1.467 (3)	C16—H16A	0.92 (3)
N9—H9A	0.83 (3)	C16—H16B	0.93 (2)
N9—H9B	0.80 (3)	C3—H3C	0.93 (2)
N9—H9C	0.99 (3)	C24—H24A	0.97 (2)
C14—C13	1.386 (2)	C24—H24B	0.97 (3)
C14—C9	1.395 (2)		
			
H2C—O2—H2D	104 (2)	C19—C18—C17	116.33 (18)
H1A—O1—H2B	106 (2)	C19—C18—H18	120.3 (14)
C15—N4—C9	108.99 (15)	C17—C18—H18	123.2 (14)
C15—N4—H4A	124.8 (16)	N2—C7—N1	109.33 (16)
C9—N4—H4A	125.4 (16)	N2—C7—C8	123.45 (17)
H3D—O3—H3E	107 (2)	N1—C7—C8	127.11 (16)
C23—N7—C17	108.55 (16)	C3—C2—C1	116.08 (18)
C23—N7—H7	126.6 (17)	C3—C2—H2A	121.8 (12)
C17—N7—H7	124.0 (17)	C1—C2—H2A	122.0 (12)
C15—N5—C14	109.14 (15)	N2—C6—C5	132.85 (18)
C15—N5—H5A	124.9 (15)	N2—C6—C1	106.35 (15)
C14—N5—H5A	125.6 (15)	C5—C6—C1	120.80 (18)
C7—N1—C1	109.27 (15)	C12—C13—C14	116.35 (17)
C7—N1—H1	125.7 (17)	C12—C13—H13	124.6 (12)
C1—N1—H1	124.5 (17)	C14—C13—H13	119.1 (12)
C7—N2—C6	109.38 (16)	N7—C23—N8	109.72 (16)
C7—N2—H2	126.4 (17)	N7—C23—C24	127.55 (18)
C6—N2—H2	123.7 (17)	N8—C23—C24	122.63 (17)
C23—N8—C22	108.85 (15)	C21—C20—C19	122.08 (18)
C23—N8—H8	123.7 (15)	C21—C20—H20	117.6 (16)
C22—N8—H8	127.0 (15)	C19—C20—H20	120.3 (15)
C16—N6—H6A	111.4 (17)	C20—C21—C22	116.12 (18)
C16—N6—H6B	115.5 (14)	C20—C21—H21	124.1 (13)
H6A—N6—H6B	104 (2)	C22—C21—H21	119.7 (13)
C16—N6—H6C	109 (2)	C13—C12—C11	122.09 (18)
H6A—N6—H6C	110 (3)	C13—C12—H12	116.6 (16)
H6B—N6—H6C	107 (2)	C11—C12—H12	121.3 (16)
C24—N9—H9A	110.4 (18)	C4—C5—C6	116.97 (18)
C24—N9—H9B	106.7 (18)	C4—C5—H5	124.2 (14)
H9A—N9—H9B	109 (2)	C6—C5—H5	118.8 (14)
C24—N9—H9C	113.6 (17)	C18—C19—C20	121.68 (18)
H9A—N9—H9C	108 (2)	C18—C19—H19	116.2 (12)
H9B—N9—H9C	109 (2)	C20—C19—H19	122.1 (12)
C8—N3—H3A	108 (2)	C5—C4—C3	121.94 (19)
C8—N3—H3B	114.2 (14)	C5—C4—H4	118.4 (13)
H3A—N3—H3B	111 (2)	C3—C4—H4	119.6 (13)
C8—N3—H3	111.0 (16)	C10—C11—C12	121.71 (19)
H3A—N3—H3	106 (3)	C10—C11—H11	115.9 (12)
H3B—N3—H3	107 (2)	C12—C11—H11	122.3 (12)
C13—C14—N5	132.44 (17)	N3—C8—C7	112.34 (16)
C13—C14—C9	121.46 (17)	N3—C8—H8A	110.4 (16)
N5—C14—C9	106.11 (15)	C7—C8—H8A	110.8 (16)
N8—C22—C17	106.45 (15)	N3—C8—H8B	109.5 (16)
N8—C22—C21	131.74 (17)	C7—C8—H8B	109.3 (16)
C17—C22—C21	121.81 (17)	H8A—C8—H8B	104 (2)
C10—C9—N4	131.77 (16)	N6—C16—C15	112.13 (17)
C10—C9—C14	122.19 (16)	N6—C16—H16A	113.4 (18)
N4—C9—C14	106.02 (15)	C15—C16—H16A	108.1 (18)
N4—C15—N5	109.73 (16)	N6—C16—H16B	108.5 (14)
N4—C15—C16	127.44 (16)	C15—C16—H16B	109.5 (15)
N5—C15—C16	122.72 (16)	H16A—C16—H16B	105 (2)
C2—C1—N1	131.96 (16)	C2—C3—C4	121.8 (2)
C2—C1—C6	122.37 (17)	C2—C3—H3C	118.8 (14)
N1—C1—C6	105.66 (16)	C4—C3—H3C	119.4 (14)
C18—C17—C22	121.97 (16)	N9—C24—C23	112.39 (17)
C18—C17—N7	131.61 (17)	N9—C24—H24A	108.5 (15)
C22—C17—N7	106.41 (15)	C23—C24—H24A	109.0 (14)
C11—C10—C9	116.19 (17)	N9—C24—H24B	105 (2)
C11—C10—H10	120.6 (12)	C23—C24—H24B	112 (2)
C9—C10—H10	123.2 (12)	H24A—C24—H24B	110 (2)
			
C15—N5—C14—C13	−179.30 (18)	C7—N2—C6—C5	178.55 (18)
C15—N5—C14—C9	0.93 (19)	C7—N2—C6—C1	−1.15 (19)
C23—N8—C22—C17	1.03 (19)	C2—C1—C6—N2	179.60 (15)
C23—N8—C22—C21	−179.49 (17)	N1—C1—C6—N2	0.52 (18)
C15—N4—C9—C10	178.27 (18)	C2—C1—C6—C5	−0.1 (3)
C15—N4—C9—C14	−0.41 (18)	N1—C1—C6—C5	−179.23 (15)
C13—C14—C9—C10	1.1 (3)	N5—C14—C13—C12	179.48 (17)
N5—C14—C9—C10	−179.15 (15)	C9—C14—C13—C12	−0.8 (2)
C13—C14—C9—N4	179.89 (15)	C17—N7—C23—N8	1.29 (19)
N5—C14—C9—N4	−0.31 (18)	C17—N7—C23—C24	−175.17 (18)
C9—N4—C15—N5	1.02 (19)	C22—N8—C23—N7	−1.5 (2)
C9—N4—C15—C16	−175.38 (18)	C22—N8—C23—C24	175.21 (16)
C14—N5—C15—N4	−1.22 (19)	C19—C20—C21—C22	0.5 (3)
C14—N5—C15—C16	175.38 (16)	N8—C22—C21—C20	179.87 (17)
C7—N1—C1—C2	−178.68 (18)	C17—C22—C21—C20	−0.7 (2)
C7—N1—C1—C6	0.28 (18)	C14—C13—C12—C11	0.2 (3)
N8—C22—C17—C18	179.76 (15)	N2—C6—C5—C4	−179.37 (18)
C21—C22—C17—C18	0.2 (2)	C1—C6—C5—C4	0.3 (3)
N8—C22—C17—N7	−0.25 (17)	C17—C18—C19—C20	−0.8 (3)
C21—C22—C17—N7	−179.79 (15)	C21—C20—C19—C18	0.3 (3)
C23—N7—C17—C18	179.37 (18)	C6—C5—C4—C3	−0.3 (3)
C23—N7—C17—C22	−0.62 (18)	C9—C10—C11—C12	0.0 (3)
N4—C9—C10—C11	−179.14 (17)	C13—C12—C11—C10	0.2 (3)
C14—C9—C10—C11	−0.6 (2)	N2—C7—C8—N3	−178.27 (17)
C22—C17—C18—C19	0.5 (2)	N1—C7—C8—N3	6.0 (3)
N7—C17—C18—C19	−179.44 (17)	N4—C15—C16—N6	−6.7 (3)
C6—N2—C7—N1	1.4 (2)	N5—C15—C16—N6	177.37 (17)
C6—N2—C7—C8	−175.02 (16)	C1—C2—C3—C4	0.1 (3)
C1—N1—C7—N2	−1.02 (19)	C5—C4—C3—C2	0.1 (3)
C1—N1—C7—C8	175.20 (17)	N7—C23—C24—N9	−8.3 (3)
N1—C1—C2—C3	178.75 (17)	N8—C23—C24—N9	175.69 (17)
C6—C1—C2—C3	−0.1 (2)		

### Hydrogen-bond geometry (Å, º)

**Table:**

*D*—H···*A*	*D*—H	H···*A*	*D*···*A*	*D*—H···*A*
O1—H1*A*···Cl4^i^	0.82 (2)	2.63 (2)	3.4431 (15)	168 (3)
O1—H2*B*···Cl2	0.81 (2)	2.36 (2)	3.1598 (18)	169 (3)
O2—H2*C*···Cl3	0.82 (2)	2.33 (2)	3.1244 (17)	165 (2)
O2—H2*D*···Cl1	0.81 (1)	2.64 (1)	3.4492 (15)	171 (3)
O3—H3*D*···Cl5^ii^	0.80 (2)	2.69 (2)	3.4586 (15)	164 (3)
O3—H3*E*···Cl6^ii^	0.82 (2)	2.32 (2)	3.1364 (18)	173 (3)
N1—H1···O1	0.83 (2)	1.92 (2)	2.746 (2)	174 (2)
N2—H2···Cl1^iii^	0.71 (2)	2.44 (2)	3.1519 (17)	175 (2)
N3—H3···Cl3	0.88 (3)	2.30 (3)	3.105 (2)	153 (2)
N3—H3*A*···Cl1	0.86 (4)	2.26 (3)	3.119 (2)	173 (3)
N3—H3*B*···Cl4^iv^	0.99 (3)	2.33 (2)	3.267 (2)	156.8 (19)
N4—H4*A*···O2	0.85 (2)	1.91 (2)	2.754 (2)	171 (2)
N5—H5*A*···Cl5	0.83 (2)	2.31 (2)	3.1205 (17)	165 (2)
N6—H6*A*···Cl2	0.87 (3)	2.33 (3)	3.107 (2)	150 (2)
N6—H6*B*···Cl1^v^	0.98 (3)	2.36 (2)	3.287 (2)	158.2 (18)
N6—H6*C*···Cl4^i^	0.79 (3)	2.35 (4)	3.1347 (19)	176 (3)
N7—H7···O3	0.85 (3)	1.89 (3)	2.742 (2)	175 (3)
N8—H8···Cl4	0.81 (2)	2.31 (2)	3.1049 (17)	172 (2)
N9—H9*A*···Cl6	0.83 (3)	2.35 (3)	3.100 (2)	151 (2)
N9—H9*B*···Cl5	0.80 (3)	2.32 (2)	3.120 (2)	176 (2)
N9—H9*C*···Cl5^vi^	0.99 (3)	2.34 (3)	3.270 (2)	157 (2)
C4—H4···Cl1^i^	0.92 (2)	2.81 (2)	3.535 (2)	136.7 (16)
C8—H8*B*···Cl3^vii^	0.93 (3)	2.65 (3)	3.544 (2)	162 (2)

Symmetry codes: (i) *x*, *y*−1, *z*; (ii) −*x*, −*y*+1, −*z*+1; (iii) −*x*+3, −*y*, −*z*+2; (iv) *x*+1, *y*−1, *z*; (v) *x*−1, *y*, *z*; (vi) −*x*+1, −*y*+1, −*z*+1; (vii) −*x*+2, −*y*, −*z*+2.
