# Crystal structure and Hirshfeld surface analysis and of 2-ammoniumylmeth­yl-1*H*-benzimidazol-3-ium chloride monohydrate. Corrigendum

**DOI:** 10.1107/S2056989018016560

**Published:** 2018-11-30

**Authors:** Pinar Sen, Sevgi Kansiz, Necmi Dege, S. Zeki Yildiz, Galyna G. Tsapyuk

**Affiliations:** a Uskudar University, Faculty of Engineering and Natural Sciences, Department of Forensic Science, 34662, Istanbul, Turkey; b Ondokuz Mayıs University, Faculty of Arts and Sciences, Department of Physics, 55139, Kurupelit, Samsun, Turkey; c Sakarya University, Faculty of Arts and Sciences, Department of Chemistry, 54187 Sakarya, Turkey; d Taras Shevchenko National University of Kyiv, Department of Chemistry, 64 Vladimirska Str., Kiev 01601, Ukraine

## Abstract

In the paper by Sen *et al.* [*Acta Cryst.* (2018), E**74**, 1517–1520], the address of the correspondence author is incorrect.

## Abstract 2

Erratum to *Acta Cryst.* (2018), E**74**, 1517–1520.

In the paper by Sen *et al.* (2018 ▸), the address of the correspondence author, Galyna G. Tsapyuk, should be ‘Taras Shevchenko National University of Kyiv, Department of Chemistry, 64 Vladimirska Str., Kiev 01601, Ukraine’, as given above.

## References

[bb1] Sen, P., Kansiz, S., Dege, N., Yildiz, S. Z. & Tsapyuk, G. G. (2018). *Acta Cryst.* E**74**, 1517–1520. 10.1107/S205698901801335X PMC6176449 30319814

